# Correction: Javaid et al. Layer-By-Layer Self-Assembled Dip Coating for Antifouling Functionalized Finishing of Cotton Textile. *Polymers* 2022, *14*, 2540

**DOI:** 10.3390/polym18141700

**Published:** 2026-07-10

**Authors:** Sana Javaid, Azhar Mahmood, Habib Nasir, Mudassir Iqbal, Naveed Ahmed, Nasir M. Ahmad

**Affiliations:** 1 School of Natural Science (SNS), National University of Science and Technology (NUST), Islamabad 44000, Pakistan; sana.javaid@sns.nust.edu.pk (S.J.); dr.azhar@sns.nust.edu.pk (A.M.); habibnasir@sns.nust.edu.pk (H.N.); mudassir.iqbal@sns.nust.edu.pk (M.I.); 2 Department of Chemistry, University of Wah, Wah Cantt 47040, Pakistan; 3 Department of Pharmacy, Quaid-i-Azam University, Islamabad 44000, Pakistan; natanoli@qau.edu.pk; 4 Polymer Research Lab, School of Chemical and Materials Engineering (SCME), National University of Sciences and Technology (NUST), Islamabad 44000, Pakistan

## Figure Legend

In the original publication [1], there was an error in the caption for “**Figure 3.** (**a**) Zeta size, (**b**) zeta potential, and (**c**) scanning electron microscope images of drug loaded nanoformulation”. The correct caption appears below. The reference [31] mentioned in the text Section 3.1 in the original publication.

**Figure 3.** (**a**) Zeta size, (**b**) Zeta potential, (**c**) scanning electron microscopy of drug loaded nanoformulation (Adopted from Ref. [31]).

The authors state that the scientific conclusions are unaffected. This correction was approved by the Academic Editor. The original publication has also been updated.
